# Supplementary material for: Nuclear corepressor SMRT acts as a strong regulator of both β-oxidation and suppressor of fibrosis in the differentiation process of mouse skeletal muscle cells
Source: PLoS One. 2022 Dec 1;17(12):e0277830. doi: 10.1371/journal.pone.0277830 (PMC9714868; doi:10.1371/journal.pone.0277830)
Supplement: S1 Table — (PDF) [file pone.0277830.s006.pdf]

S1 Table : Key Resources

Oligonucleotides

sgRNAs	Sequence
mSmrt-A1s	CACCGAAAATGTGAGTCACTGCTCT
mSmrt-A1r	AAACAGAGCAGTGACTCACATTTTC
mSmrt-ABC2s	CACCGACTGGCGTGTGTCAGACGGC
mSmrt-ABC2r	AAACGCCGTCTGACACACGCCAGTC

Primers for screening and DNA sequnece	Sequence
SMRT CRISPR Exon2 screening F	CTGCTTCTCCCTGTGTTCTCC
SMRT CRISPR Exon2 screening R	AAGTGTGCCCAGAGCAGTC

Mouse Gene Name targeted by si-RNA	Source	Identifier (Assay ID)
Stealth siRNAs : Tgf-β1	Thermo Fisher Scientific	Tgfb1MSS211649
Stealth siRNAs: Tgf-β3	Thermo Fisher Scientific	Tgfb3MSS211655
Stealth RNAi siRNA Negative Control HiGC	Thermo Fisher Scientific	12935400

Primers for reverse transcription quantitative polymerase chain reaction RT- qPCR analyses

Mouse Gene Name	Primer Sequence	Mouse Gene Name	Primer Sequence
Smrt	(F) ATGGCTTGTCTGAGCAGGAG	Hdac3	(F) GCATTCGAGGACATGGGGAA
	(R) GGGTCATCCATGAGTCCATT		(R) TTTCGGACAGTGTAGCCACC
MyoD	(F) CATCCAGCCCGCGCTCCAAC	Ncor1	(F) AGAAGCAGGTCAGCAGCAGT
	(R) GGGCCGCTGTAATCCATCATGCC		(R) TGGAGGAGGCCCTTTATCTT
Myogenin	(F) CAGTGAATGCAACTCCCACAG	Tgf-β1	(F) ATGGCTTGTCTGAGCAGGAG
	(R) TGGACGTAAGGGAGTGCAGA		(R) GGGTCATCCATGAGTCCATT
Myomaker	(F) ATCGCTACCAAGAGGCGTT	Tgf-β2	(F) GCTAATGTTGTTGCCCTCCT
	(R) CACAGCACAGACAAACCAGG		(R) GCAGCAATTATCCTGCACATT
Myomerger (Long form)	(F) ACCAGCTTTCATGCCAGAAG	Tgf-β3	(F) CGAGTGGCTGTTGAGGAGA
	(R) ATGTCTTGGGAGCTCAGTCG		(R) GCTGAAAGGTGTGACATGGA
Myomerger (Short form)	(F) CAGGAGGGCAAGAAGTTCAG	Bmp4	(F) GAGGAGTTTCCATCACGAAGA
	(R) ATGTCTTGGGAGCTCAGTCG		(R) GCTCTGCCGAGGAGATCA
Myh4	(F) GCAGGACTTGGTGGACAAAC	Ctgf	(F) CCACCCGAGTTACCAATGAC
	(R) ACTTGGCCAGGTTGACATTG		(R) GCTTGGCGATTTTAGGTGTC
Pparδ	(F) GCTGCTGCAGAAGATGGCA	Fgf2	(F) AAGCGGCTCTACTGCAAGAA
	(R) CACTGCATCATCTGGGCATG		(R) GTAACACACTTAGAAGCCAGCAG
Pgc-1α	(F) AACCAGTACAACAATGAGCCTG	Col1a1	(F) ATGTTCAGCTTTGTGGACCT
	(R) AATGAGGGCAATCCGTCTTCA		(R) CAGCTGACTTCAGGGATGT
Ampk2	(F) TCGCAGACAGCCCCAAAG	Smad2	(F) ATGTCGTCCATCTTGCCATTC
	(R) TTGGGCTTCGTTGTGTTGAG		(R) AACCGTCCTGTTTTCTTTAGCTT
Ucp2	(F) CTACAAGACCATTGCACGAGAGG	Smad3	(F) CCCCAGCACATAATAACTTGG
	(R) AGCTGCTCATAGGTGACAAACAT		(R) AGGAGATGGAGCACCAGAAG
Ucp3	(F) CTGCACCGCCAGATGAGTTT	β-Catenin	(F) AGACAGCTCGTTGTACTGCT
	(R) ATCATGGCTTGAAATCGGACC		(R) GTGTCGTGATGGCGTAGAAC
Cpt-1β	(F) CCCATGTGCTCCTACCAGAT	Alk5	(F) CATCAGGGTCTGGATCAGGTT
	(R) CCTTGAAGAAGCGACCTTTG		(R) GTAACACAATGGTCCTGGCAA
Cd36	(F) GCCCAATGGAGCCATCTTTG	Gapdh	(F) TGTGTCCGTCGTGGATCTGA
	(R) AGCTGCTACAGCCAGATTCA		(R) TTGCTGTTGAAGTCGAGGAG

Antibodies

Name	Source	Identifier (Catalog #)
Anti-SMRTe Antibody	Merck-Millipore	Cat.#06-891
Anti-Myosin Heavy Chain (clone MF20) Purified	Invitrogen	14-6503-82
Anti-RNA Polymerase II, CTD, clone 8WG16	Merck-Millipore	Cat.#05-952-I-25-UG
PPAR delta Polyclonal Antibody	Invitrogen	PA1-823A
Anti-PGC-1, C-Terminal (777-797) Rabbit pAb	Merck-Millipore	516557
AMPK Alpha2 Rabbit Polyclonal antibody	Proteintech Group Inc.	18167-1-AP
Anti GAPDH, Monoclonal Antibody, Peroxidase Conjugated	Fujifilm-Wako	Code no. 015-25473
Anti-HDAC3, clone 3G6	Merck-Millipore	Cat.#05-813
Anti-acetyl-Histone H4 (Lys5)	Merck-Millipore	Cat.#07-327
Histone H4 Rabbit Polyclonal antibody	Proteintech Group Inc.	16047-1-AP
NCoR Antibody	Bethyl Lab. Inc.	A301-145A
Collagen I alpha 1 Antibody	Novus Bio.	NBP1-30054
Anti-FGF-2 Antibody (G2)	Santa Cruz Bio. Inc.	sc-365106
Anti-BMP4 antibody	Abcam	ab39973
Smad3 (C67H9) Rabbit mAb	Cell Signaling Technology	#9523
Phospho-Smad3 (Ser423/425) (C25A9) Rabbit mAb	Cell Signaling Technology	#9520
Anti-Smad2 antibody	Abcam	ab33875
Anti-Smad2 (phospho S467) antibody	Abcam	ab280888
Anti-β-catenin Antibody (E-5)	Santa Cruz Bio. Inc.	sc-7963
Anti-TGF-beta Receptor, type I Polyclonal Antibody	Merck-Millipore	Cat.#ABF17-I
Alexa Fluor 488 goat anti-mouse IgG (H+L)	Invitrogen	A11029
Goat anti-Rabbit IgG (H+L) Secondary Antibody, HRP	Invitrogen	Cat.#65-6120
ECL Anti-mouse IgG, HRP-linked whole antibody (from sheep)	Cytiva	NA931V
