# Supplementary material for: Nuclear corepressor SMRT acts as a strong regulator of both β-oxidation and suppressor of fibrosis in the differentiation process of mouse skeletal muscle cells
Source: PLoS One. 2022 Dec 1;17(12):e0277830. doi: 10.1371/journal.pone.0277830 (PMC9714868; doi:10.1371/journal.pone.0277830)
Supplement: S1 Raw images — (PDF) [file pone.0277830.s008.pdf]

Fig1C SMRT

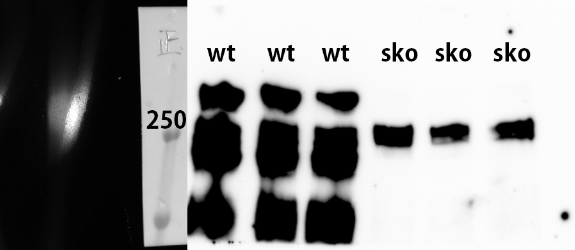

Signal was detected using ChemiDoc™ Touch Imaging System and quantified by
Image Lab™ Touch Software (BIO-RAD).
The signal image was captured by chemiluminescent blot.

Precision Plus Protein™ Standards Dual Color (BIO-RAD) was used as a protein marker.
The marker image was captured by Ponceau S blot concurrently with the signal image.
The sectional image of protein marker is displayed in the left side of corresponding signal image.

Fig1C MYH4

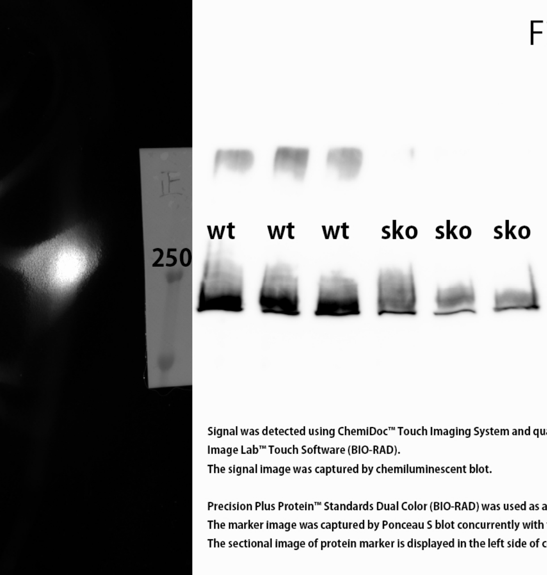

Signal was detected using ChemiDoc™ Touch Imaging System and quantified by
Image Lab™ Touch Software (BIO-RAD).
The signal image was captured by chemiluminescent blot.

Precision Plus Protein™ Standards Dual Color (BIO-RAD) was used as a protein marker.
The marker image was captured by Ponceau S blot concurrently with the signal image.
The sectional image of protein marker is displayed in the left side of corresponding signal image.

Fig1C POL2

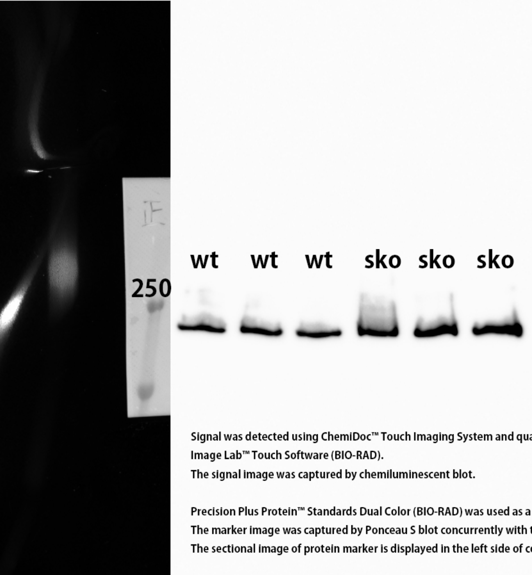

Signal was detected using ChemiDoc™ Touch Imaging System and quantified by Image Lab™ Touch Software (BIO-RAD).
The signal image was captured by chemiluminescent blot.

Precision Plus Protein™ Standards Dual Color (BIO-RAD) was used as a protein marker.
The marker image was captured by Ponceau S blot concurrently with the signal image.
The sectional image of protein marker is displayed in the left side of corresponding signal image.

Fig2B PPARdelta

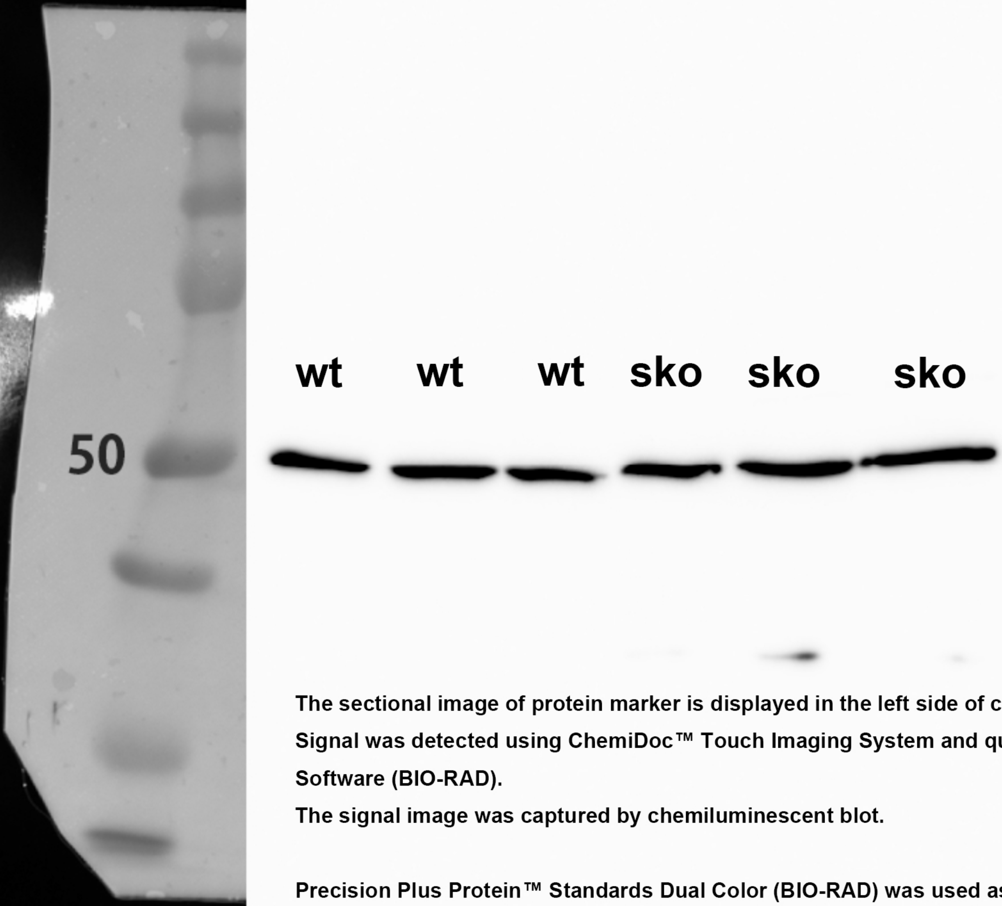

The sectional image of protein marker is displayed in the left side of corresponding signal image.

Signal was detected using ChemiDoc™ Touch Imaging System and quantified by Image Lab™ Touch Software (BIO-RAD).

The signal image was captured by chemiluminescent blot.

Precision Plus Protein™ Standards Dual Color (BIO-RAD) was used as a protein marker.

The marker image was captured by Ponceau S blot concurrently with the signal image.

Fig2B PGC-1alpha

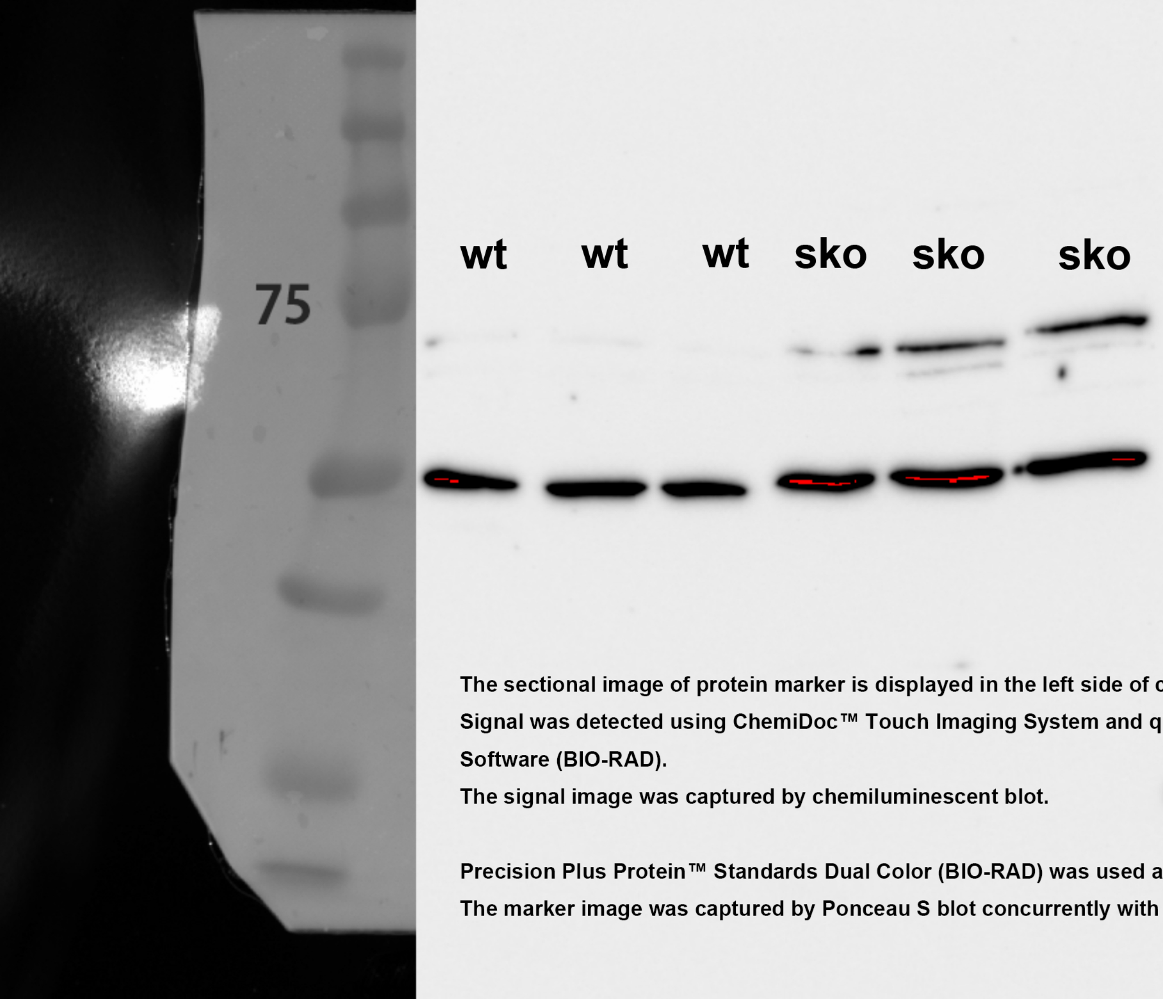

The sectional image of protein marker is displayed in the left side of corresponding signal image.
Signal was detected using ChemiDoc™ Touch Imaging System and quantified by Image Lab™ Touch Software (BIO-RAD).
The signal image was captured by chemiluminescent blot.

Precision Plus Protein™ Standards Dual Color (BIO-RAD) was used as a protein marker.
The marker image was captured by Ponceau S blot concurrently with the signal image.

Fig2B AMPK2

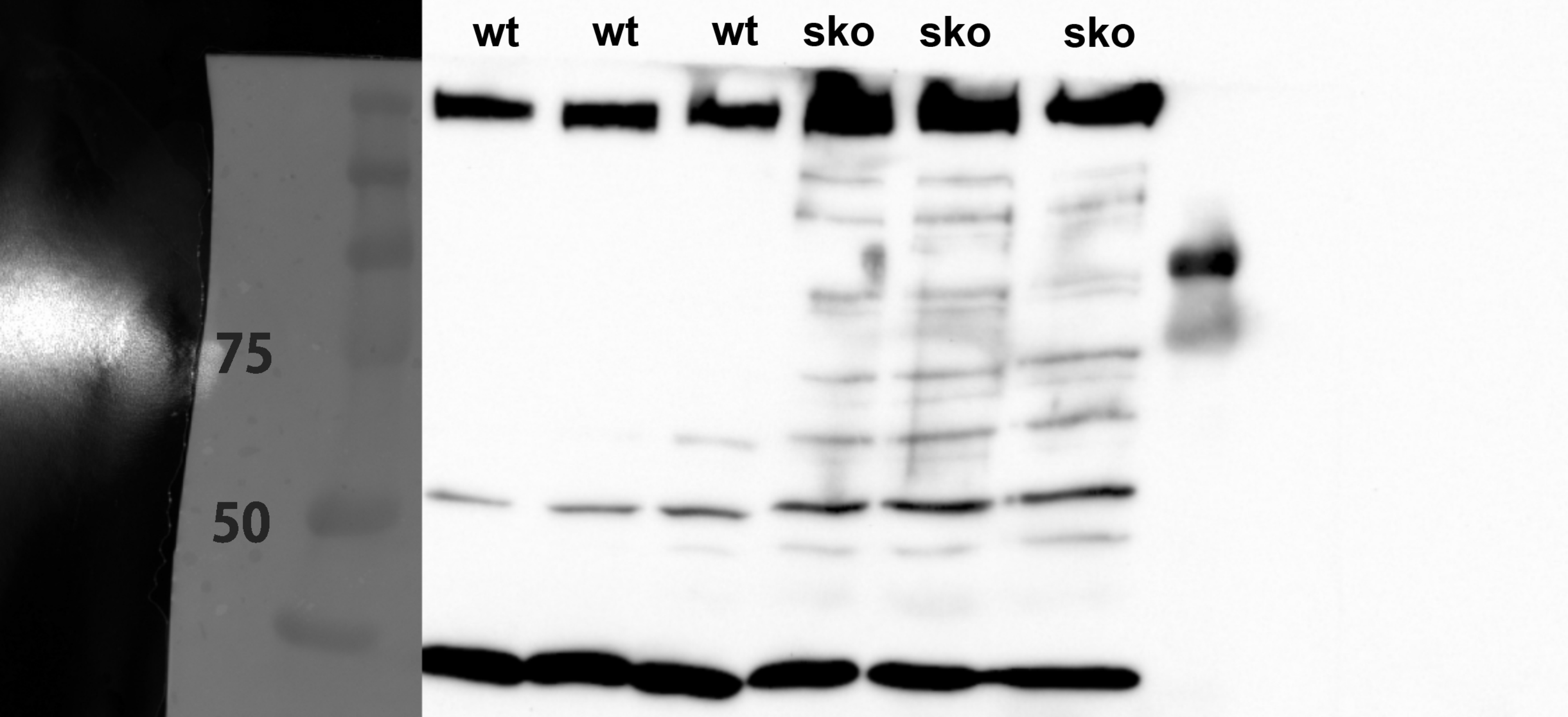

The sectional image of protein marker is displayed in the left side of corresponding signal image.
Signal was detected using ChemiDoc™ Touch Imaging System and quantified by Image Lab™ Touch Software (BIO-RAD).
The signal image was captured by chemiluminescent blot.

Precision Plus Protein™ Standards Dual Color (BIO-RAD) was used as a protein marker.
The marker image was captured by Ponceau S blot concurrently with the signal image.

Fig2B GAPDH

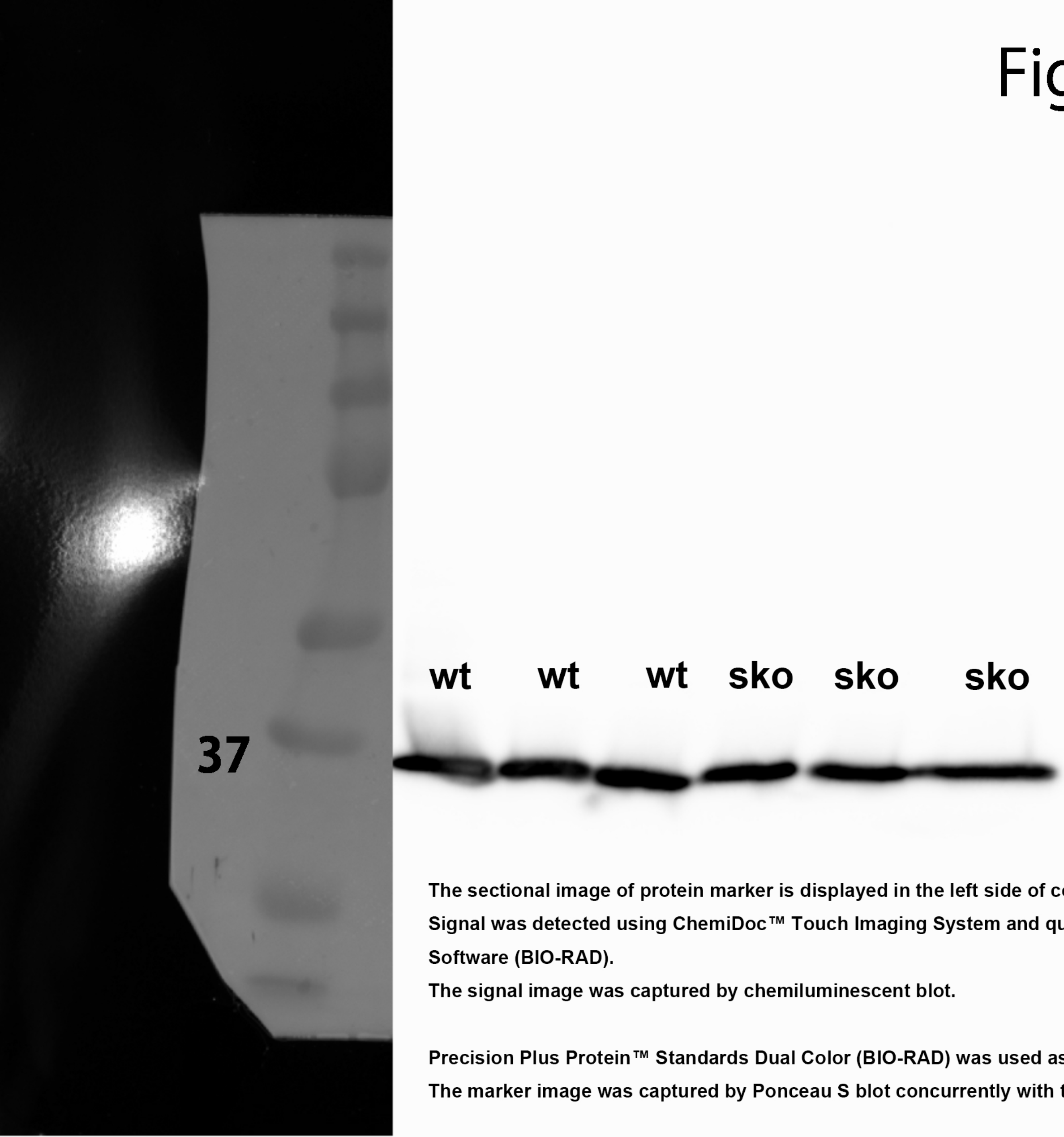

The sectional image of protein marker is displayed in the left side of corresponding signal image.
Signal was detected using ChemiDoc™ Touch Imaging System and quantified by Image Lab™ Touch Software (BIO-RAD).
The signal image was captured by chemiluminescent blot.

Precision Plus Protein™ Standards Dual Color (BIO-RAD) was used as a protein marker.
The marker image was captured by Ponceau S blot concurrently with the signal image.

Fig2D HDAC3

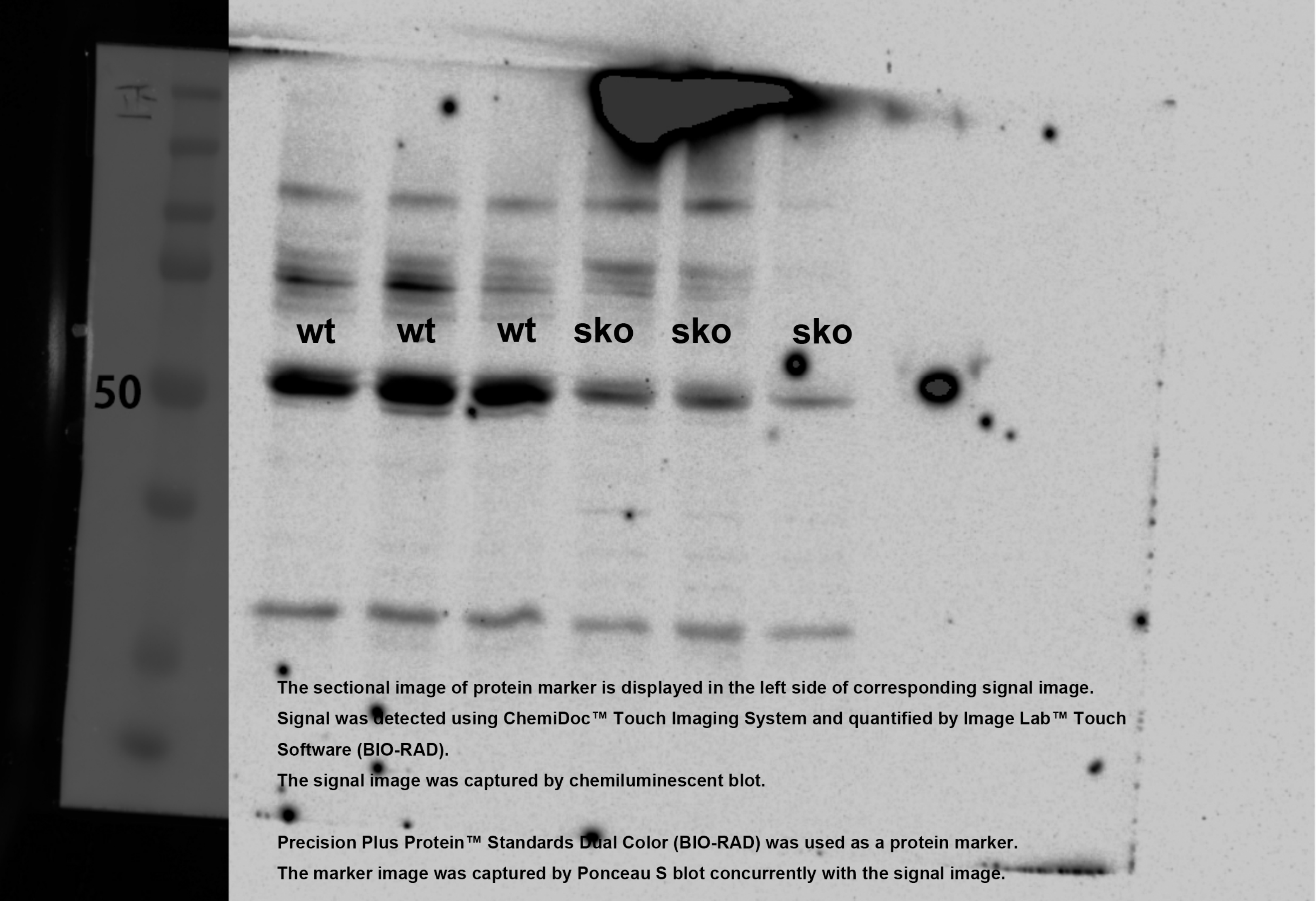

The sectional image of protein marker is displayed in the left side of corresponding signal image.

Signal was detected using ChemiDoc™ Touch Imaging System and quantified by Image Lab™ Touch Software (BIO-RAD).

The signal image was captured by chemiluminescent blot.

Precision Plus Protein™ Standards Dual Color (BIO-RAD) was used as a protein marker.

The marker image was captured by Ponceau S blot concurrently with the signal image.

Fig2D GAPDH

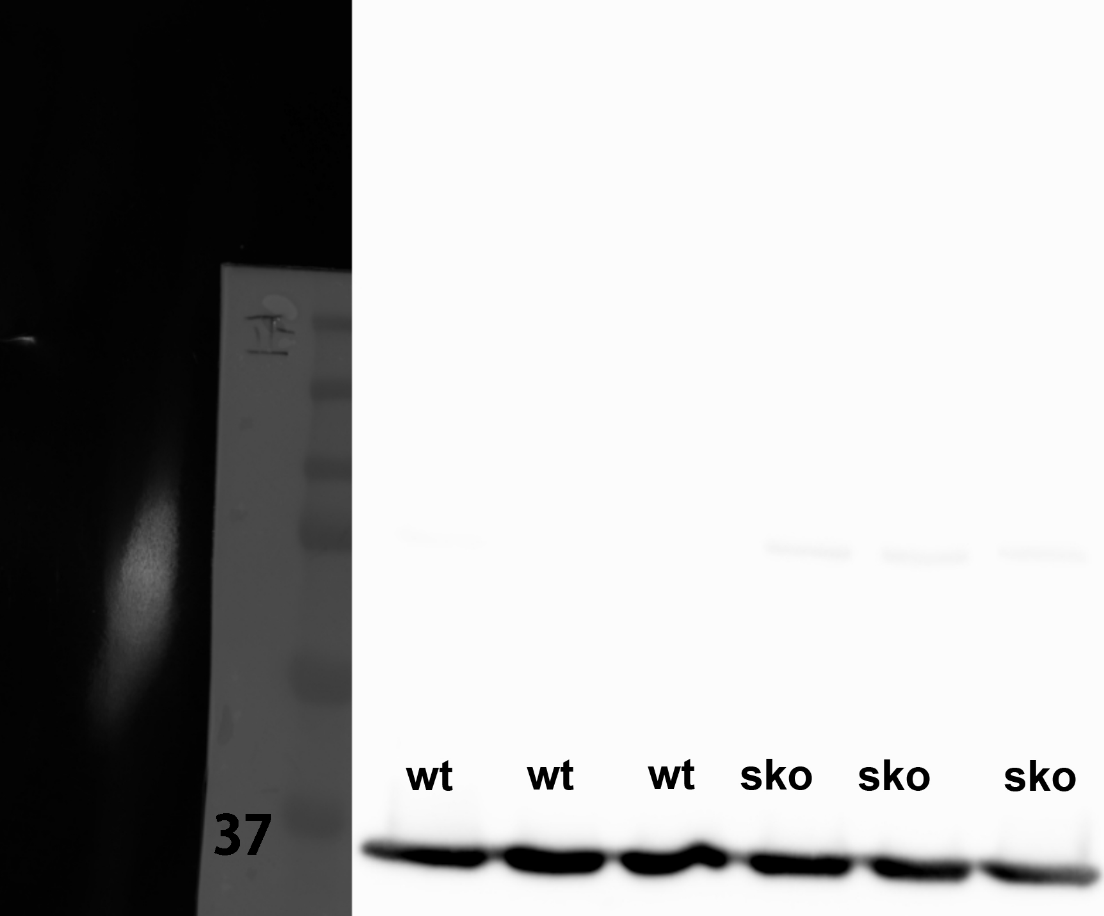

The sectional image of protein marker is displayed in the left side of corresponding signal image.
Signal was detected using ChemiDoc™ Touch Imaging System and quantified by Image Lab™ Touch Software (BIO-RAD).
The signal image was captured by chemiluminescent blot.

Precision Plus Protein™ Standards Dual Color (BIO-RAD) was used as a protein marker.
The marker image was captured by Ponceau S blot concurrently with the signal image.

Fig2E H4K5ac

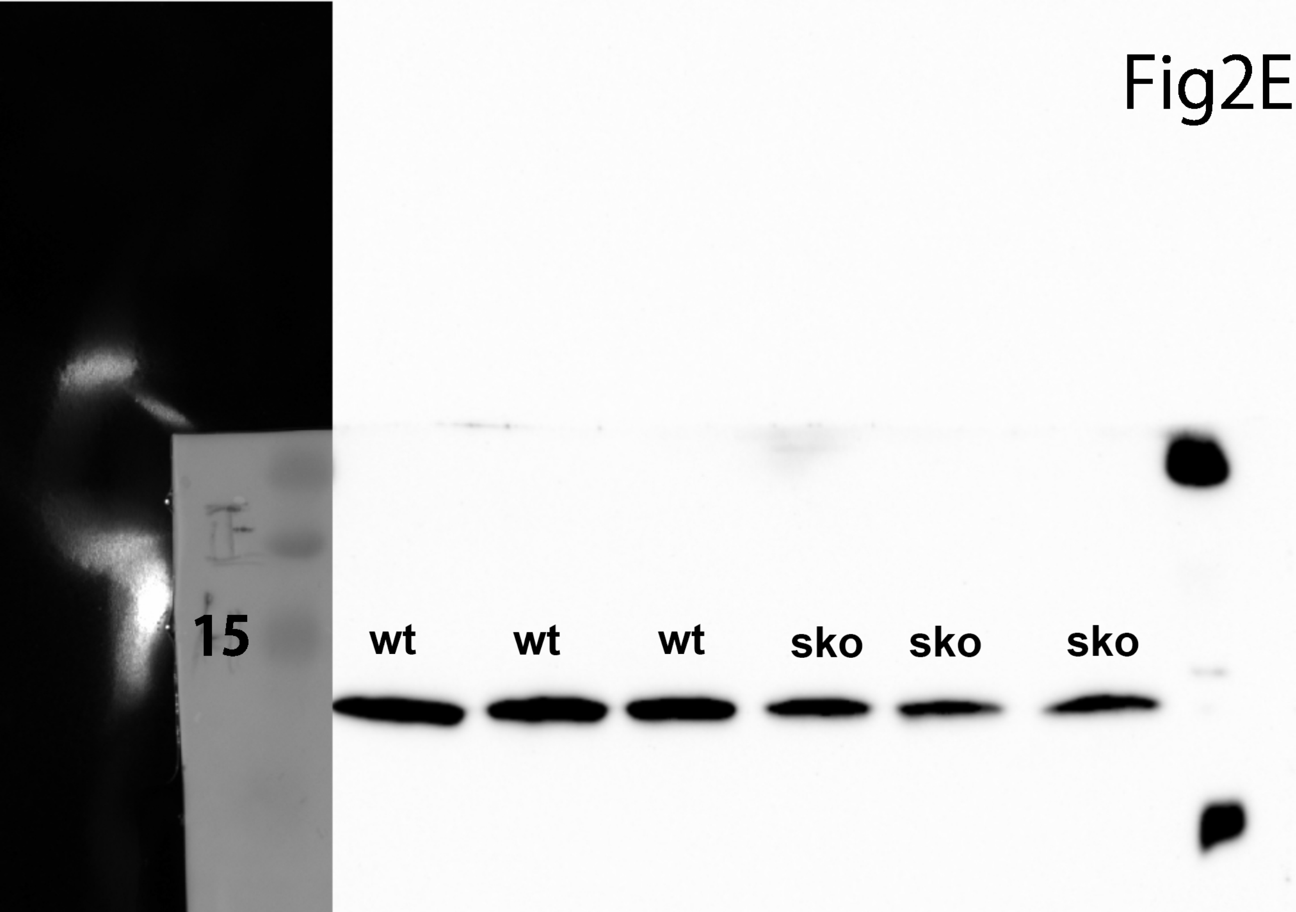

The sectional image of protein marker is displayed in the left side of corresponding signal image.
Signal was detected using ChemiDoc™ Touch Imaging System and quantified by Image Lab™ Touch Software (BIO-RAD).
The signal image was captured by chemiluminescent blot.

Precision Plus Protein™ Standards Dual Color (BIO-RAD) was used as a protein marker.
The marker image was captured by Ponceau S blot concurrently with the signal image.

Fig2E H4

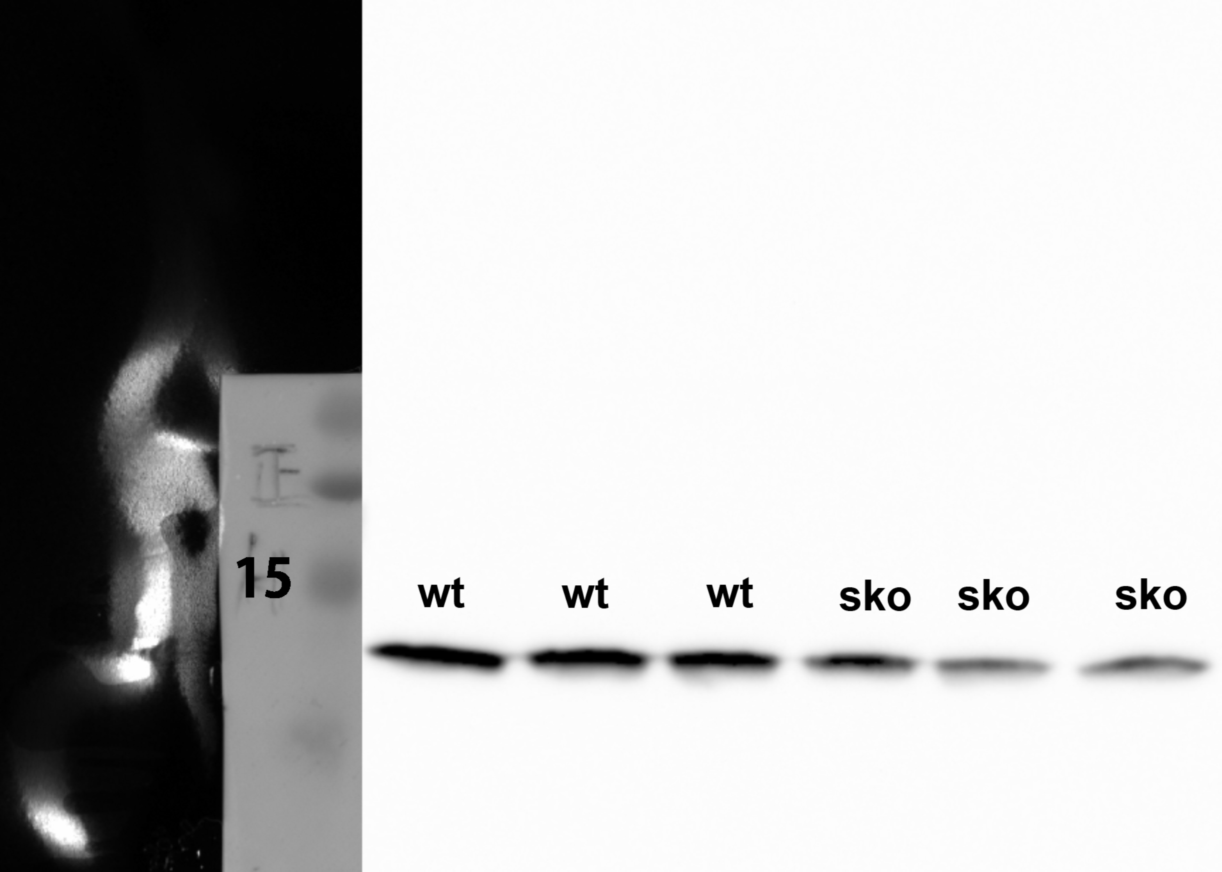

The sectional image of protein marker is displayed in the left side of corresponding signal image.
Signal was detected using ChemiDoc™ Touch Imaging System and quantified by Image Lab™ Touch Software (BIO-RAD).
The signal image was captured by chemiluminescent blot.

Precision Plus Protein™ Standards Dual Color (BIO-RAD) was used as a protein marker.
The marker image was captured by Ponceau S blot concurrently with the signal image.

Fig3C COL1A1

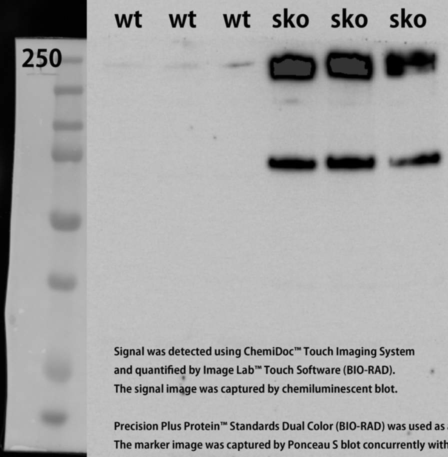

Signal was detected using ChemiDoc™ Touch Imaging System
and quantified by Image Lab™ Touch Software (BIO-RAD).
The signal image was captured by chemiluminescent blot.

Precision Plus Protein™ Standards Dual Color (BIO-RAD) was used as a protein marker.
The marker image was captured by Ponceau S blot concurrently with the signal image.
The sectional image of protein marker is displayed in the left side of corresponding signal image.

Fig3C FGF2 isoform

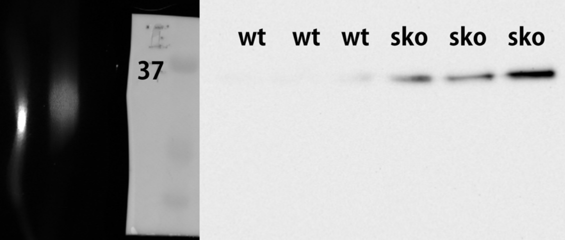

Signal was detected using ChemiDoc™ Touch Imaging System
and quantified by Image Lab™ Touch Software (BIO-RAD).
The signal image was captured by chemiluminescent blot.

Precision Plus Protein™ Standards Dual Color (BIO-RAD) was used as a protein marker.
The marker image was captured by Ponceau S blot concurrently with the signal image.
The sectional image of protein marker is displayed in the left side of corresponding signal image.

Fig3C GAPDH

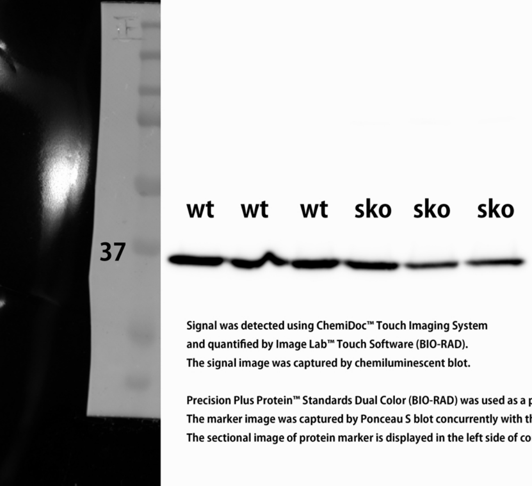

Signal was detected using ChemiDoc™ Touch Imaging System
and quantified by Image Lab™ Touch Software (BIO-RAD).
The signal image was captured by chemiluminescent blot.

Precision Plus Protein™ Standards Dual Color (BIO-RAD) was used as a protein marker.
The marker image was captured by Ponceau S blot concurrently with the signal image.
The sectional image of protein marker is displayed in the left side of corresponding signal image.

Fig3C BMP4

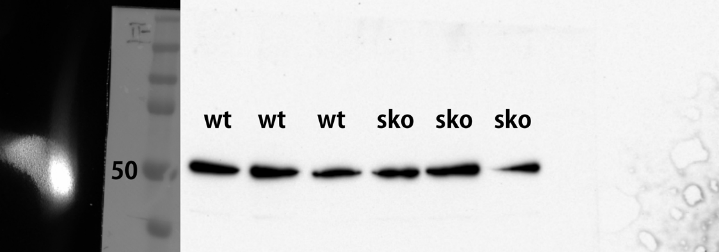

Signal was detected using ChemiDoc™ Touch Imaging System
and quantified by Image Lab™ Touch Software (BIO-RAD).
The signal image was captured by chemiluminescent blot.

Precision Plus Protein™ Standards Dual Color (BIO-RAD) was used as a protein marker.
The marker image was captured by Ponceau S blot concurrently with the signal image.
The sectional image of protein marker is displayed in the left side of corresponding signal image.

Fig3C GAPDH

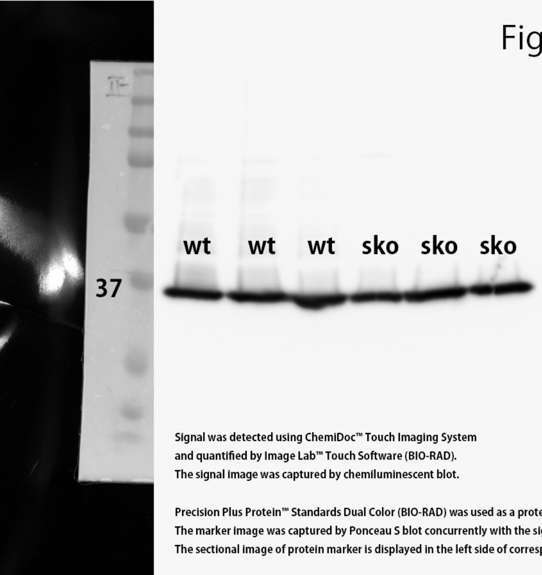

Signal was detected using ChemiDoc™ Touch Imaging System
and quantified by Image Lab™ Touch Software (BIO-RAD).
The signal image was captured by chemiluminescent blot.

Precision Plus Protein™ Standards Dual Color (BIO-RAD) was used as a protein marker.
The marker image was captured by Ponceau S blot concurrently with the signal image.
The sectional image of protein marker is displayed in the left side of corresponding signal image.

Fig3E SMAD2

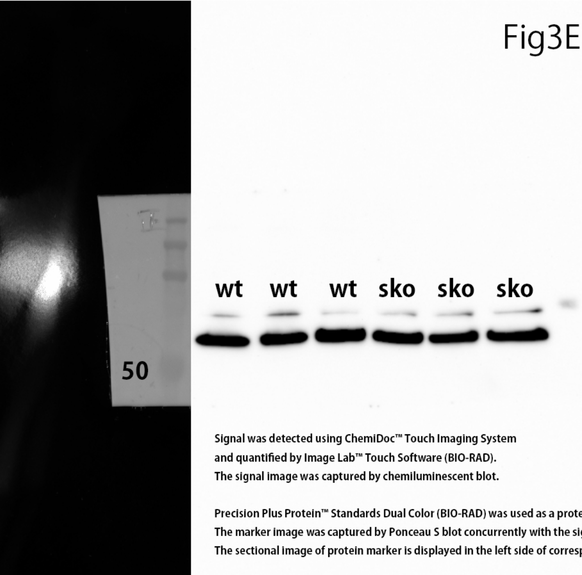

Signal was detected using ChemiDoc™ Touch Imaging System
and quantified by Image Lab™ Touch Software (BIO-RAD).
The signal image was captured by chemiluminescent blot.

Precision Plus Protein™ Standards Dual Color (BIO-RAD) was used as a protein marker.
The marker image was captured by Ponceau S blot concurrently with the signal image.
The sectional image of protein marker is displayed in the left side of corresponding signal image.

Fig3E p-SMAD2

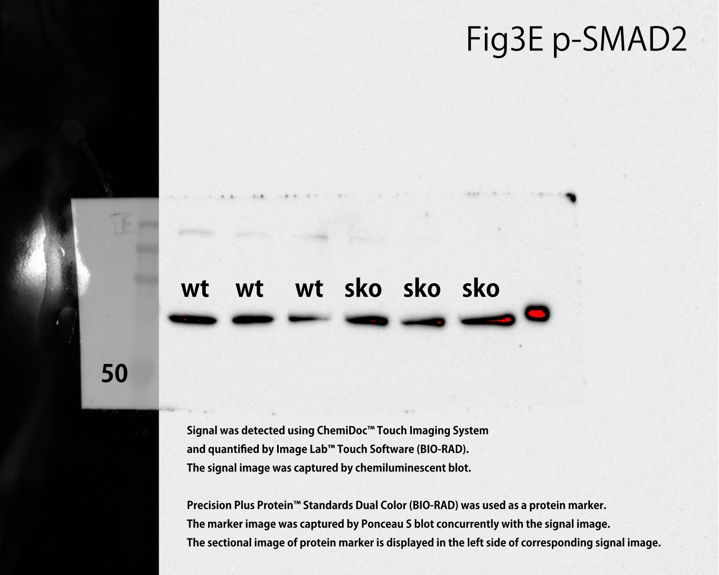

Signal was detected using ChemiDoc™ Touch Imaging System and quantified by Image Lab™ Touch Software (BIO-RAD). The signal image was captured by chemiluminescent blot.

Precision Plus Protein™ Standards Dual Color (BIO-RAD) was used as a protein marker. The marker image was captured by Ponceau S blot concurrently with the signal image. The sectional image of protein marker is displayed in the left side of corresponding signal image.

Fig3E SMAD3

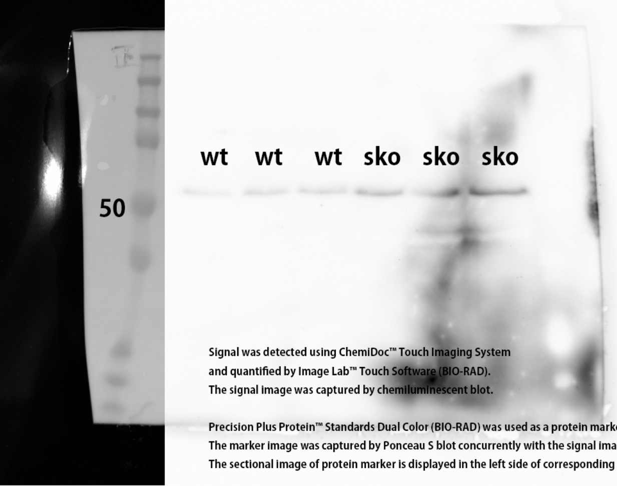

Signal was detected using ChemiDoc™ Touch Imaging System
and quantified by Image Lab™ Touch Software (BIO-RAD).
The signal image was captured by chemiluminescent blot.

Precision Plus Protein™ Standards Dual Color (BIO-RAD) was used as a protein marker.
The marker image was captured by Ponceau S blot concurrently with the signal image.
The sectional image of protein marker is displayed in the left side of corresponding signal image.

Fig3E p-SMAD3

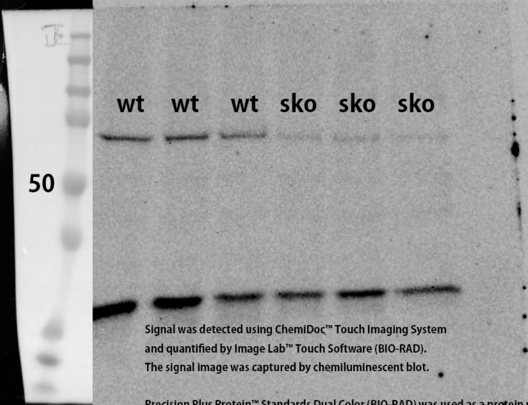

Signal was detected using ChemiDoc™ Touch Imaging System and quantified by Image Lab™ Touch Software (BIO-RAD).
The signal image was captured by chemiluminescent blot.

Precision Plus Protein™ Standards Dual Color (BIO-RAD) was used as a protein marker.
The marker image was captured by Ponceau S blot concurrently with the signal image.
The sectional image of protein marker is displayed in the left side of corresponding signal image.

Fig3E beta-Catenin

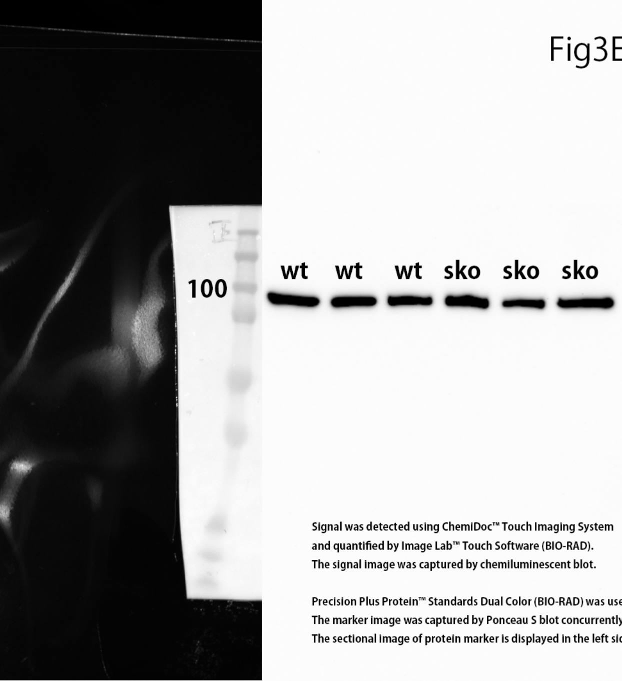

Signal was detected using ChemiDoc™ Touch Imaging System
and quantified by Image Lab™ Touch Software (BIO-RAD).
The signal image was captured by chemiluminescent blot.

Precision Plus Protein™ Standards Dual Color (BIO-RAD) was used as a protein marker.
The marker image was captured by Ponceau S blot concurrently with the signal image.
The sectional image of protein marker is displayed in the left side of corresponding signal image.

Fig3E GAPDH

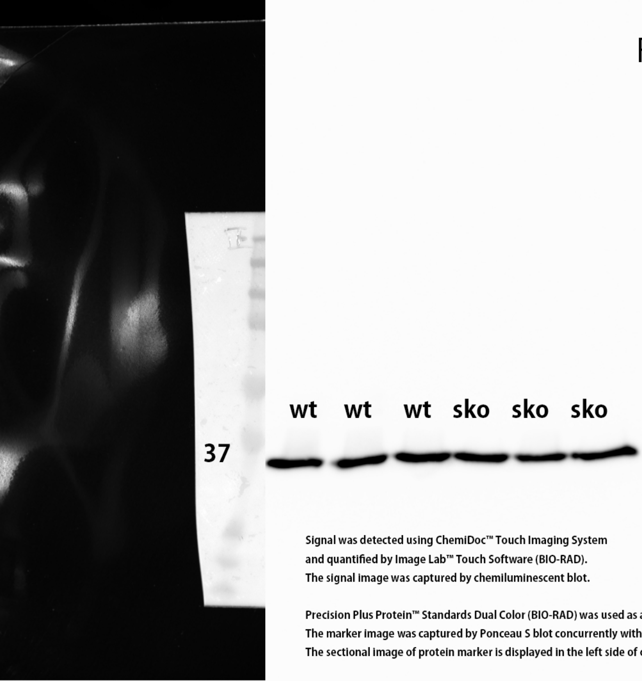

Signal was detected using ChemiDoc™ Touch Imaging System
and quantified by Image Lab™ Touch Software (BIO-RAD).
The signal image was captured by chemiluminescent blot.

Precision Plus Protein™ Standards Dual Color (BIO-RAD) was used as a protein marker.
The marker image was captured by Ponceau S blot concurrently with the signal image.
The sectional image of protein marker is displayed in the left side of corresponding signal image.

Fig4C MYH4

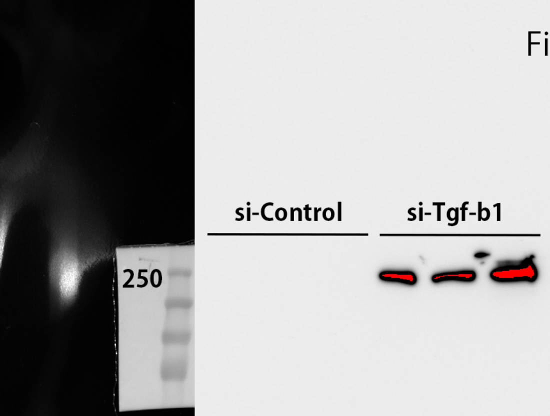

Signal was detected using ChemiDoc™ Touch Imaging System
and quantified by Image Lab™ Touch Software (BIO-RAD).
The signal image was captured by chemiluminescent blot.

Precision Plus Protein™ Standards Dual Color (BIO-RAD) was used as a protein marker.
The marker image was captured by Ponceau S blot concurrently with the signal image.
The sectional image of protein marker is displayed in the left side of corresponding signal image.

Fig4C COL1A1

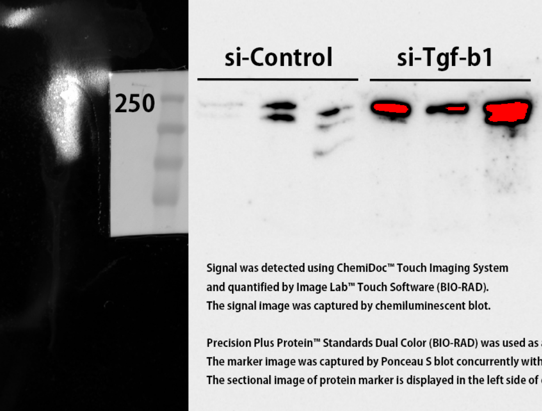

Signal was detected using ChemiDoc™ Touch Imaging System
and quantified by Image Lab™ Touch Software (BIO-RAD).
The signal image was captured by chemiluminescent blot.

Precision Plus Protein™ Standards Dual Color (BIO-RAD) was used as a protein marker.
The marker image was captured by Ponceau S blot concurrently with the signal image.
The sectional image of protein marker is displayed in the left side of corresponding signal image.

Fig4C POL2

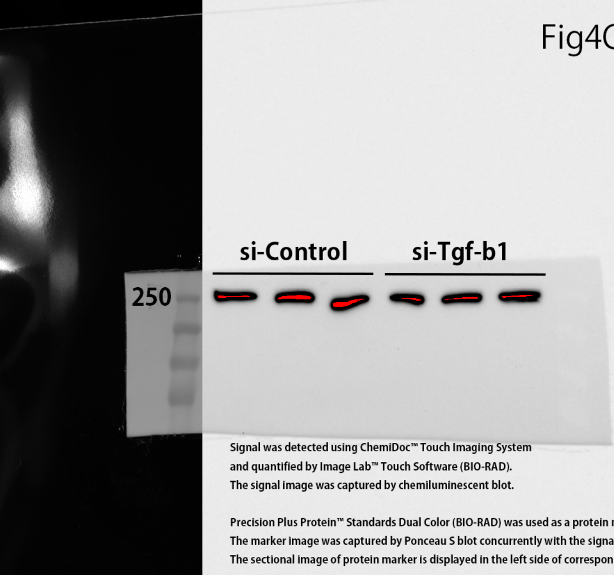

Signal was detected using ChemiDoc™ Touch Imaging System
and quantified by Image Lab™ Touch Software (BIO-RAD).
The signal image was captured by chemiluminescent blot.

Precision Plus Protein™ Standards Dual Color (BIO-RAD) was used as a protein marker.
The marker image was captured by Ponceau S blot concurrently with the signal image.
The sectional image of protein marker is displayed in the left side of corresponding signal image.

Fig4F MYH4

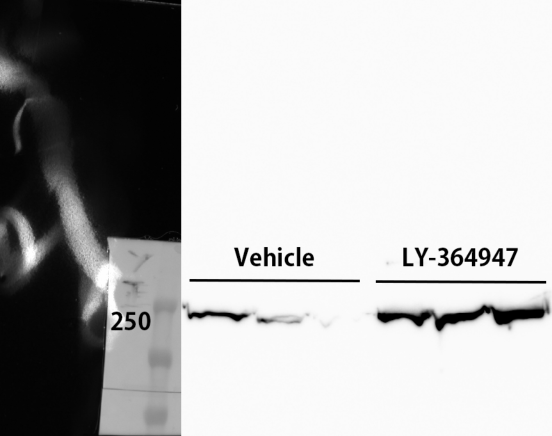

Signal was detected using ChemiDoc™ Touch Imaging System
and quantified by Image Lab™ Touch Software (BIO-RAD).
The signal image was captured by chemiluminescent blot.

Precision Plus Protein™ Standards Dual Color (BIO-RAD) was used as a protein marker.
The marker image was captured by Ponceau S blot concurrently with the signal image.
The sectional image of protein marker is displayed in the left side of corresponding signal image.

Fig4F COL1A1

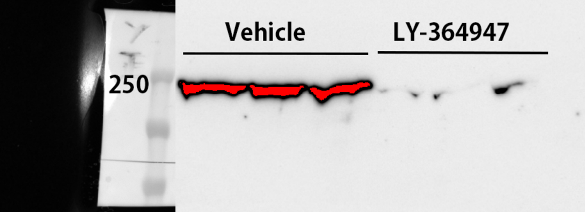

Signal was detected using ChemiDoc™ Touch Imaging System
and quantified by Image Lab™ Touch Software (BIO-RAD).
The signal image was captured by chemiluminescent blot.

Precision Plus Protein™ Standards Dual Color (BIO-RAD) was used as a protein marker.
The marker image was captured by Ponceau S blot concurrently with the signal image.
The sectional image of protein marker is displayed in the left side of corresponding signal image.

Fig4F POL2

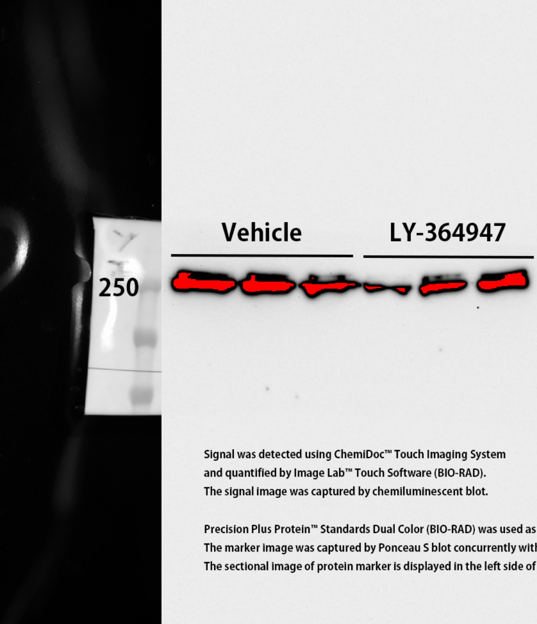

Signal was detected using ChemiDoc™ Touch Imaging System
and quantified by Image Lab™ Touch Software (BIO-RAD).
The signal image was captured by chemiluminescent blot.

Precision Plus Protein™ Standards Dual Color (BIO-RAD) was used as a protein marker.
The marker image was captured by Ponceau S blot concurrently with the signal image.
The sectional image of protein marker is displayed in the left side of corresponding signal image.

Fig4G p-SMAD2

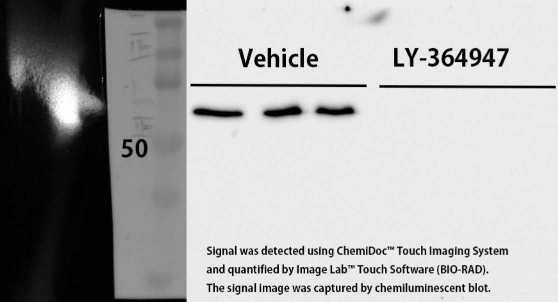

Signal was detected using ChemiDoc™ Touch Imaging System
and quantified by Image Lab™ Touch Software (BIO-RAD).
The signal image was captured by chemiluminescent blot.

Precision Plus Protein™ Standards Dual Color (BIO-RAD) was used as a protein marker.
The marker image was captured by Ponceau S blot concurrently with the signal image.
The sectional image of protein marker is displayed in the left side of corresponding signal image.

Fig4G p-SMAD3

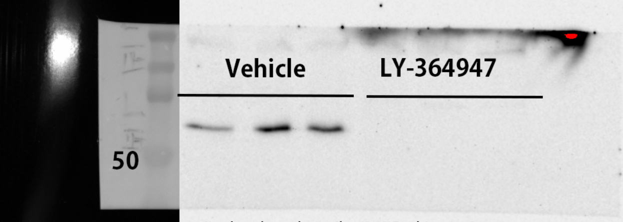

Signal was detected using ChemiDoc™ Touch Imaging System and quantified by Image Lab™ Touch Software (BIO-RAD).
The signal image was captured by chemiluminescent blot.

Precision Plus Protein™ Standards Dual Color (BIO-RAD) was used as a protein marker.
The marker image was captured by Ponceau S blot concurrently with the signal image.
The sectional image of protein marker is displayed in the left side of corresponding signal image.

Fig4G GAPDH

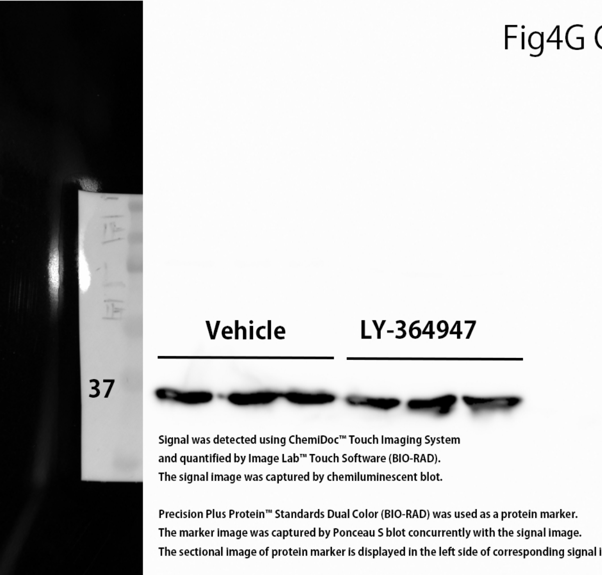

Signal was detected using ChemiDoc™ Touch Imaging System and quantified by Image Lab™ Touch Software (BIO-RAD).
The signal image was captured by chemiluminescent blot.

Precision Plus Protein™ Standards Dual Color (BIO-RAD) was used as a protein marker.
The marker image was captured by Ponceau S blot concurrently with the signal image.
The sectional image of protein marker is displayed in the left side of corresponding signal image.

Fig4H PPAR delta

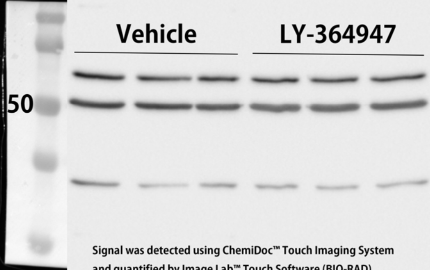

Signal was detected using ChemiDoc™ Touch Imaging System
and quantified by Image Lab™ Touch Software (BIO-RAD).
The signal image was captured by chemiluminescent blot.

Precision Plus Protein™ Standards Dual Color (BIO-RAD) was used as a protein marker.
The marker image was captured by Ponceau S blot concurrently with the signal image.
The sectional image of protein marker is displayed in the left side of corresponding signal image.

Fig4H PGC-1 alpha

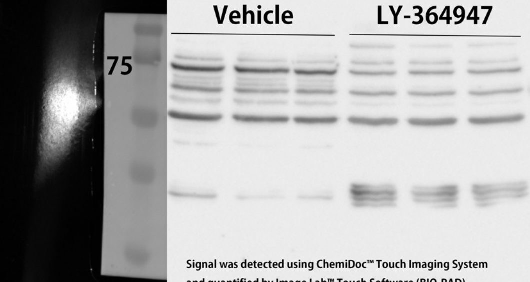

Signal was detected using ChemiDoc™ Touch Imaging System
and quantified by Image Lab™ Touch Software (BIO-RAD).
The signal image was captured by chemiluminescent blot.

Precision Plus Protein™ Standards Dual Color (BIO-RAD) was used as a protein marker.
The marker image was captured by Ponceau S blot concurrently with the signal image.
The sectional image of protein marker is displayed in the left side of corresponding signal image.

Fig4H AMPK2

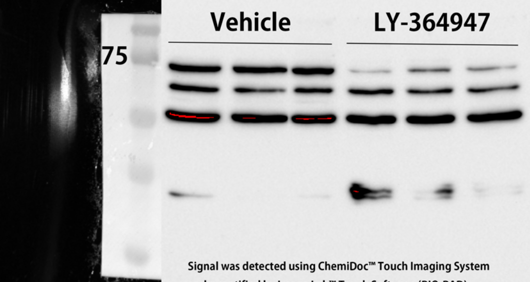

Signal was detected using ChemiDoc™ Touch Imaging System
and quantified by Image Lab™ Touch Software (BIO-RAD).
The signal image was captured by chemiluminescent blot.

Precision Plus Protein™ Standards Dual Color (BIO-RAD) was used as a protein marker.
The marker image was captured by Ponceau S blot concurrently with the signal image.
The sectional image of protein marker is displayed in the left side of corresponding signal image.

Fig4H GAPDH

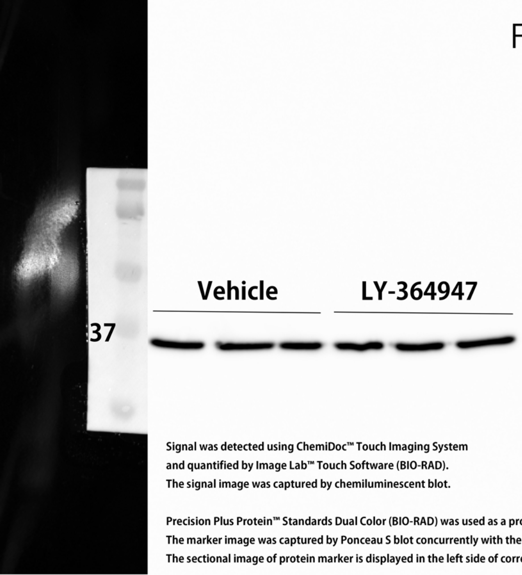

Signal was detected using ChemiDoc™ Touch Imaging System
and quantified by Image Lab™ Touch Software (BIO-RAD).
The signal image was captured by chemiluminescent blot.

Precision Plus Protein™ Standards Dual Color (BIO-RAD) was used as a protein marker.
The marker image was captured by Ponceau S blot concurrently with the signal image.
The sectional image of protein marker is displayed in the left side of corresponding signal image.

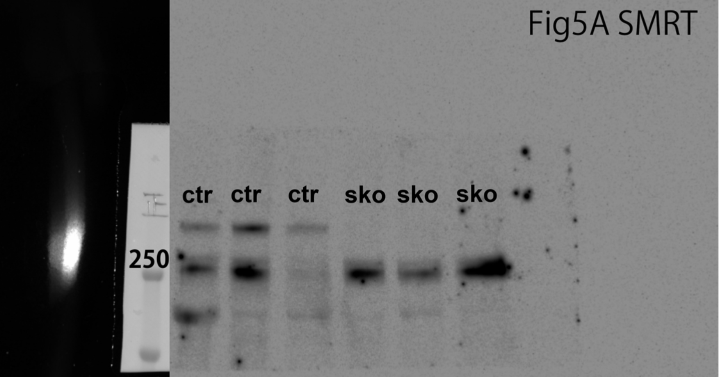

Signal was detected using ChemiDoc™ Touch Imaging System and quantified by Image Lab™ Touch Software (BIO-RAD).

The signal image was captured by chemiluminescent blot.

Precision Plus Protein™ Standards Dual Color (BIO-RAD) was used as a protein marker.

The marker image was captured by Ponceau S blot concurrently with the signal image.

The sectional image of protein marker is displayed in the left side of corresponding signal image.

Fig5A POL2

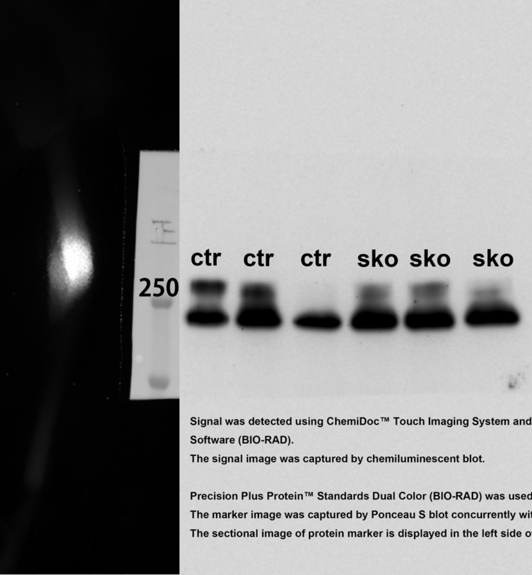

Signal was detected using ChemiDoc™ Touch Imaging System and quantified by Image Lab™ Touch Software (BIO-RAD).

The signal image was captured by chemiluminescent blot.

Precision Plus Protein™ Standards Dual Color (BIO-RAD) was used as a protein marker.

The marker image was captured by Ponceau S blot concurrently with the signal image.

The sectional image of protein marker is displayed in the left side of corresponding signal image.

Fig5C PPAR delta

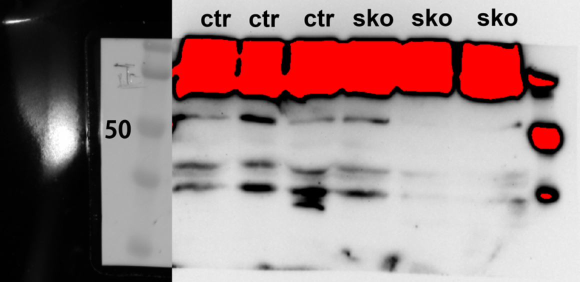

Signal was detected using ChemiDoc™ Touch Imaging System and quantified by Image Lab™ Touch Software (BIO-RAD).
The signal image was captured by chemiluminescent blot.

Precision Plus Protein™ Standards Dual Color (BIO-RAD) was used as a protein marker.
The marker image was captured by Ponceau S blot concurrently with the signal image.
The sectional image of protein marker is displayed in the left side of corresponding signal image.

Fig5C PGC-1 alpha

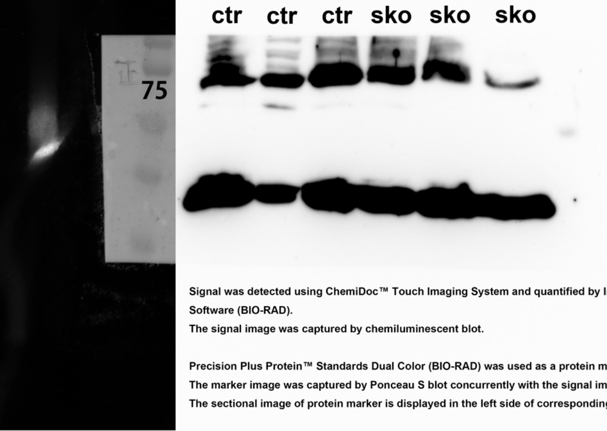

Signal was detected using ChemiDoc™ Touch Imaging System and quantified by Image Lab™ Touch Software (BIO-RAD).

The signal image was captured by chemiluminescent blot.

Precision Plus Protein™ Standards Dual Color (BIO-RAD) was used as a protein marker.

The marker image was captured by Ponceau S blot concurrently with the signal image.

The sectional image of protein marker is displayed in the left side of corresponding signal image.

Fig5C GAPDH

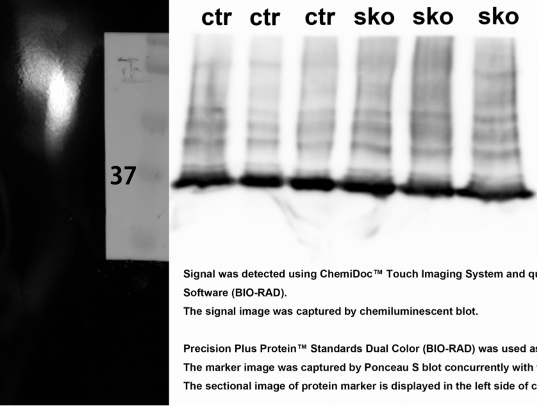

Signal was detected using ChemiDoc™ Touch Imaging System and quantified by Image Lab™ Touch Software (BIO-RAD).

The signal image was captured by chemiluminescent blot.

Precision Plus Protein™ Standards Dual Color (BIO-RAD) was used as a protein marker.

The marker image was captured by Ponceau S blot concurrently with the signal image.

The sectional image of protein marker is displayed in the left side of corresponding signal image.

S1 Fig B SMRT

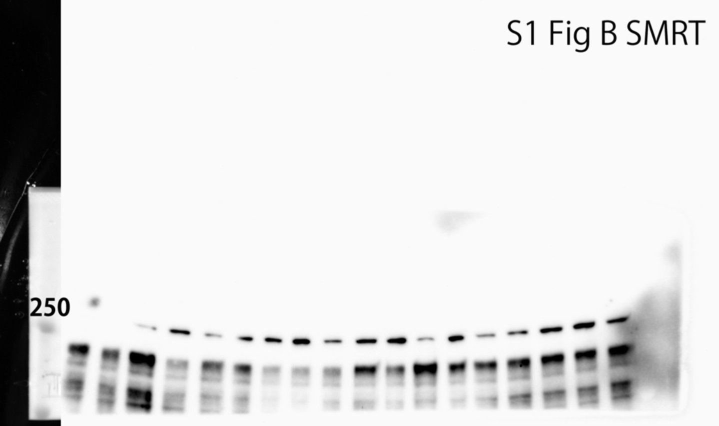

Signal was detected using ChemiDoc™ Touch Imaging System
and quantified by Image Lab™ Touch Software (BIO-RAD).
The signal image was captured by chemiluminescent blot.

Precision Plus Protein™ Standards Dual Color (BIO-RAD) was used as a protein marker.
The marker image was captured by Ponceau S blot concurrently with the signal image.
The sectional image of protein marker is displayed in the left side of corresponding signal image.

S1 FigB POL2

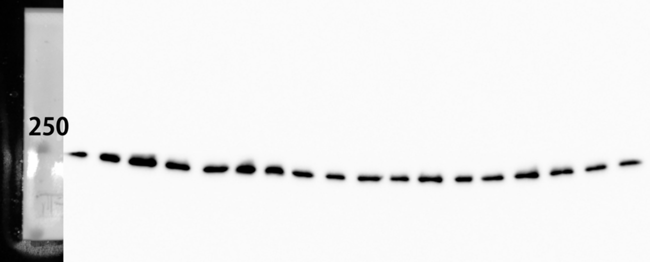

Signal was detected using ChemiDoc™ Touch Imaging System and quantified by Image Lab™ Touch Software (BIO-RAD).
The signal image was captured by chemiluminescent blot.

Precision Plus Protein™ Standards Dual Color (BIO-RAD) was used as a protein marker.
The marker image was captured by Ponceau S blot concurrently with the signal image.
The sectional image of protein marker is displayed in the left side of corresponding signal image.

S1 FigE SMRT

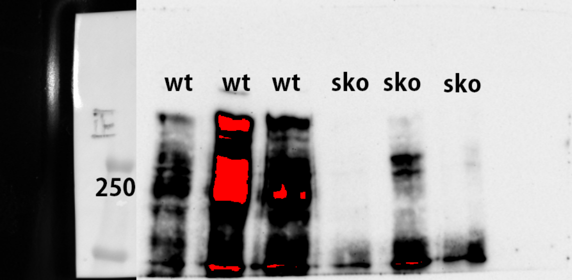

Signal was detected using ChemiDoc™ Touch Imaging System and quantified by Image Lab™ Touch Software (BIO-RAD)
The signal image was captured by chemiluminescent blot.

Precision Plus Protein™ Standards Dual Color (BIO-RAD) was used as a protein marker.
The marker image was captured by Ponceau S blot concurrently with the signal image.
The sectional image of protein marker is displayed in the left side of corresponding signal image.

S1 FigE MYH4

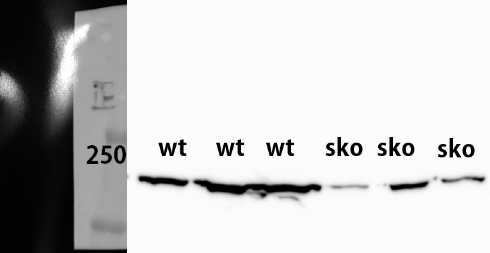

Signal was detected using ChemiDoc™ Touch Imaging System and quantified by Image Lab™ Touch Software (BIO-RAD)
The signal image was captured by chemiluminescent blot.

Precision Plus Protein™ Standards Dual Color (BIO-RAD) was used as a protein marker.
The marker image was captured by Ponceau S blot concurrently with the signal image.
The sectional image of protein marker is displayed in the left side of corresponding signal image.

S1 FigE POL2

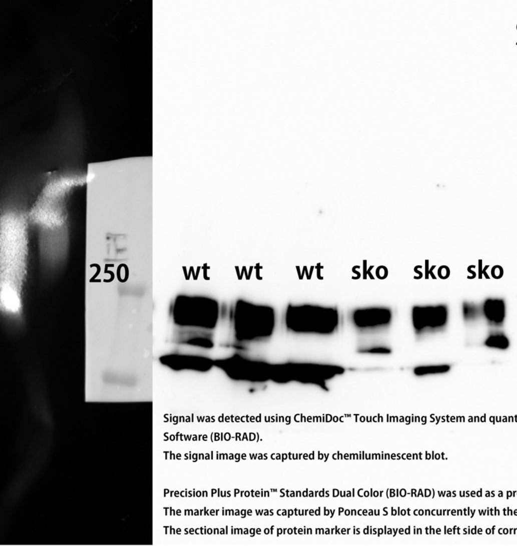

Signal was detected using ChemiDoc™ Touch Imaging System and quantified by Image Lab™ Touch Software (BIO-RAD).
The signal image was captured by chemiluminescent blot.

Precision Plus Protein™ Standards Dual Color (BIO-RAD) was used as a protein marker.
The marker image was captured by Ponceau S blot concurrently with the signal image.
The sectional image of protein marker is displayed in the left side of corresponding signal image.

S2 FigB PPAR delta

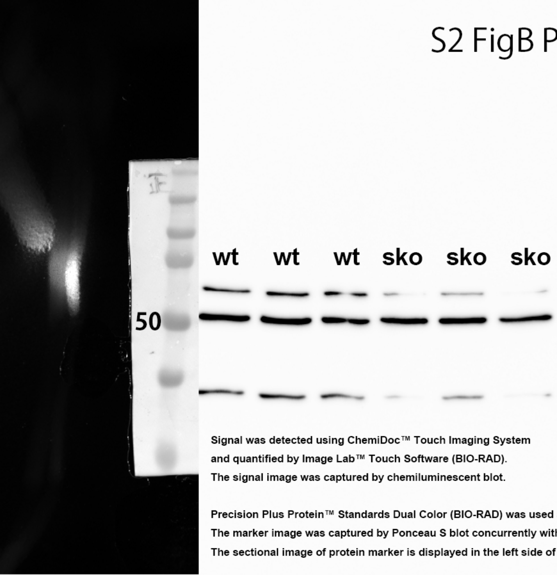

Signal was detected using ChemiDoc™ Touch Imaging System
and quantified by Image Lab™ Touch Software (BIO-RAD).
The signal image was captured by chemiluminescent blot.

Precision Plus Protein™ Standards Dual Color (BIO-RAD) was used as a protein marker.
The marker image was captured by Ponceau S blot concurrently with the signal image.
The sectional image of protein marker is displayed in the left side of corresponding signal image.

S2 FigB PGC-1alpha

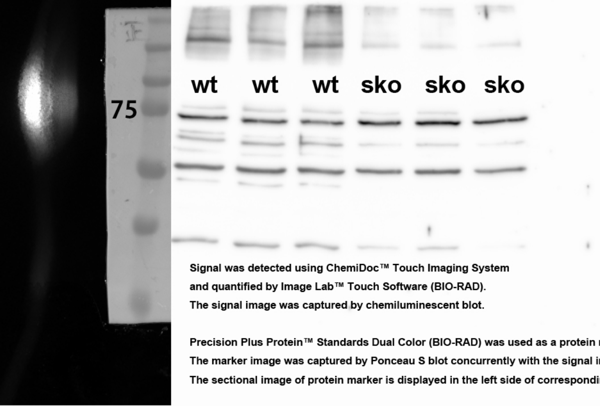

Signal was detected using ChemiDoc™ Touch Imaging System
and quantified by Image Lab™ Touch Software (BIO-RAD).
The signal image was captured by chemiluminescent blot.

Precision Plus Protein™ Standards Dual Color (BIO-RAD) was used as a protein marker.
The marker image was captured by Ponceau S blot concurrently with the signal image.
The sectional image of protein marker is displayed in the left side of corresponding signal image.

S2 FigB GAPDH

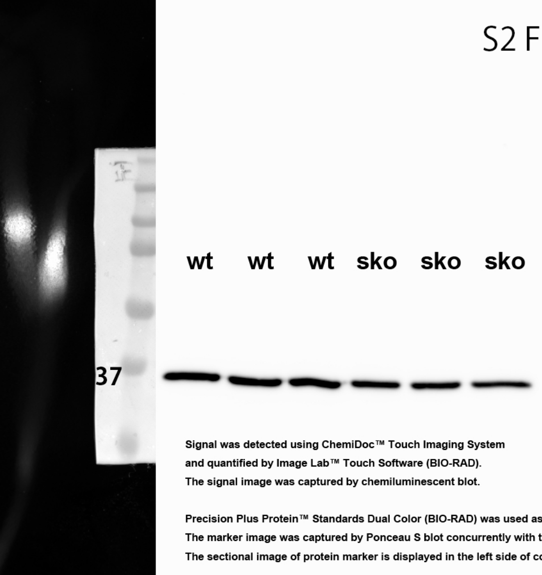

Signal was detected using ChemiDoc™ Touch Imaging System
and quantified by Image Lab™ Touch Software (BIO-RAD).
The signal image was captured by chemiluminescent blot.

Precision Plus Protein™ Standards Dual Color (BIO-RAD) was used as a protein marker.
The marker image was captured by Ponceau S blot concurrently with the signal image.
The sectional image of protein marker is displayed in the left side of corresponding signal image.

S2 FigE NCoR1

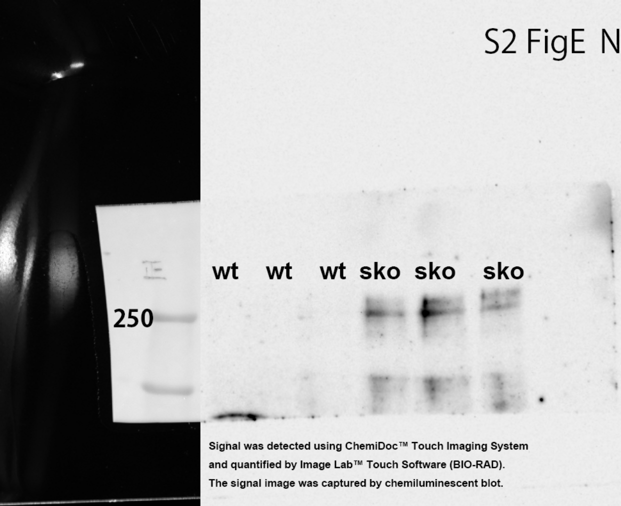

Signal was detected using ChemiDoc™ Touch Imaging System and quantified by Image Lab™ Touch Software (BIO-RAD).
The signal image was captured by chemiluminescent blot.

Precision Plus Protein™ Standards Dual Color (BIO-RAD) was used as a protein marker.
The marker image was captured by Ponceau S blot concurrently with the signal image.
The sectional image of protein marker is displayed in the left side of corresponding signal image.

S2 FigE POL2

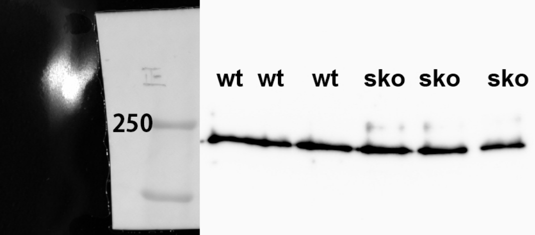

Signal was detected using ChemiDoc™ Touch Imaging System
and quantified by Image Lab™ Touch Software (BIO-RAD).
The signal image was captured by chemiluminescent blot.

Precision Plus Protein™ Standards Dual Color (BIO-RAD) was used as a protein marker.
The marker image was captured by Ponceau S blot concurrently with the signal image.
The sectional image of protein marker is displayed in the left side of corresponding signal image.

S4 FigC MYH4

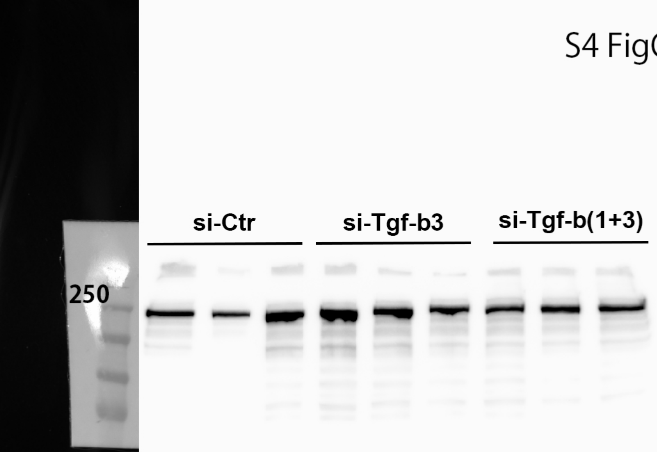

Signal was detected using ChemiDoc™ Touch Imaging System
and quantified by Image Lab™ Touch Software (BIO-RAD).
The signal image was captured by chemiluminescent blot.

Precision Plus Protein™ Standards Dual Color (BIO-RAD) was used as a protein marker.
The marker image was captured by Ponceau S blot concurrently with the signal image.
The sectional image of protein marker is displayed in the left side of corresponding signal image.

S4 FigC COL1A1

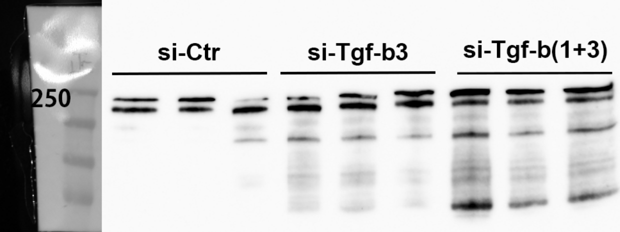

Signal was detected using ChemiDoc™ Touch Imaging System
and quantified by Image Lab™ Touch Software (BIO-RAD).
The signal image was captured by chemiluminescent blot.

Precision Plus Protein™ Standards Dual Color (BIO-RAD) was used as a protein marker.
The marker image was captured by Ponceau S blot concurrently with the signal image.
The sectional image of protein marker is displayed in the left side of corresponding signal image.

S4 FigC POL2

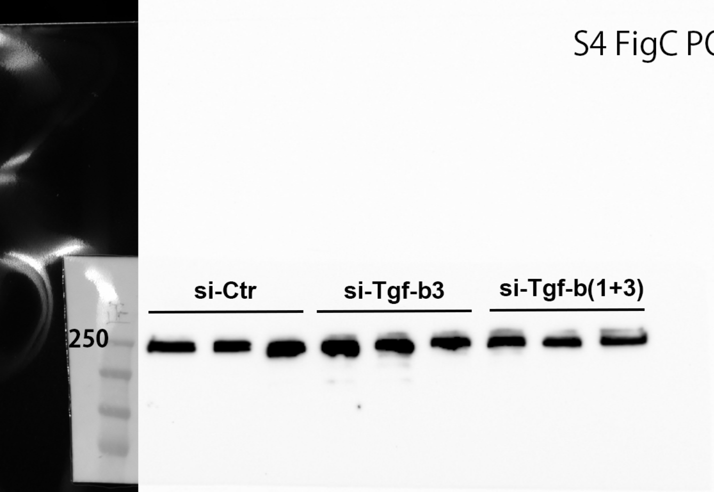

Signal was detected using ChemiDoc™ Touch Imaging System
and quantified by Image Lab™ Touch Software (BIO-RAD).
The signal image was captured by chemiluminescent blot.

Precision Plus Protein™ Standards Dual Color (BIO-RAD) was used as a protein marker.
The marker image was captured by Ponceau S blot concurrently with the signal image.
The sectional image of protein marker is displayed in the left side of corresponding signal image.
